# Tackling medical student stress: beyond individual resilience

**DOI:** 10.1007/s40037-015-0190-z

**Published:** 2015-05-28

**Authors:** Christopher Watling

**Affiliations:** Postgraduate Medical Education, Schulich School of Medicine and Dentistry, Medical Sciences Building, Room M103, Western University, N6A 5C1 London, ON Canada

Stress plagues medical students, and risks sapping the motivation and compassion we so badly need them to maintain. Recognizing this risk, medical educators have responded with interventions that enhance students’ coping and time management skills and bolster their psychological supports. Offices of learner wellness have sprung up at medical schools around the world. Why, then, do surveys continue to highlight alarming levels of stress and burnout among students [1]? Kotter et al.’s study of stressors and health-promoting interventions at a German medical school, although modest in scale, usefully redirects our thinking away from individual students and towards setting and system issues [2].

In Kotter’s study, curricular stressors dominated student focus group discussions. Inadequate prioritization of course material, unbalanced workload distribution, long working hours in some courses, and burdensome examination scheduling contributed to student stress. Students’ suggestions for health promoting interventions focused more on reducing sources of stress than on boosting their own coping skills. They proposed modifications to the stress-inducing elements of the curriculum, including redistributing the workload, prioritizing the content, emphasizing clinical relevance, and reconsidering the grading system [2].

Students’ experiences of stress are shaped by both internal and external influences. Individual vulnerability to the negative effects of stress interacts with curricular, organizational, and institutional factors [3]. Despite the recognized role of the educational environment in creating student stress, however, most interventions have been aimed at the individual student. Educators are encouraged to foster resilience, teach coping strategies, and ingrain habits of self-care [4]. But, as Dyrbye has noted, medical schools have a responsibility that extends beyond promoting self-care, and includes establishing the kinds of learning environments that support student wellbeing [5]. Individual approaches to stress reduction are thus essential, but insufficient.

Medicine’s historical tendency to build stress-resistant individuals rather than to build wellness-supporting environments may reflect the values of the profession. Stress permeates medical practice, and so some stress is necessary in training. Moderate amounts of stress may in fact enhance performance [6]. We must be sure about what we are trying to accomplish with health-promoting programmes within medical schools; abolishing stress is neither achievable nor desirable [7]. Indeed, students must learn to shoulder the stress of the profession with a resilience that preserves their humanity and compassion. But is surviving a stressful educational curriculum the best route to resilience? Many studies suggest the opposite. Instead of creating individuals who can navigate stress with their humanity intact, our curricula are fostering depression, anxiety, and burnout in our students [8].

Curricula do not simply materialize. We build them, brick by brick, on a foundation of deliberate pedagogical decisions. And we can rebuild them when cracks in the foundation appear. Reconsidering long-endorsed pedagogical choices can reap significant wellness rewards. Several studies have shown, for example, that a move from a graded to a pass-fail assessment system reduces medical student stress without compromising their performance on exit examinations [9, 10]. And Kiessling et al. have demonstrated that student-centred curriculum reform reduces stress while enhancing feelings of support among students [11].

Medical schools should look not only to their formal curriculum design when considering the wellbeing of their students, but also to their hidden curricula. Some authors have asserted that a hidden curriculum that stigmatizes mental illness is covertly discouraging students from seeking help, undermining wellness promoting efforts [5]. Kotter et al. highlight another hidden curricular influence—the potential for the intolerance of absences in medical school courses to reinforce a problematic culture of presenteeism among graduate doctors [2]. High rates of presenteeism among residents have been identified, suggesting that working despite personal illness may reflect ingrained values in the profession [12]. A simple curricular decision about absence rules may not only create unnecessary stress for students, but also lay the groundwork for later behaviour that undercuts both self-care and patient care priorities.

Learning is not simply an individual journey; rather, it is a process of enculturation. We need medical students to learn habits of self-care so they can sustain careers focused on caring for others. How do we enculturate self-care? Not by offering a slate of wellness activities for individual students without simultaneously attending to the way the learning environment may corrode student well-being. By turning our attention to how our curricular decisions shape student wellbeing, we have an opportunity for meaningful progress on the problem of student stress.

## References

[1] Chang E, Eddins-Folensbee F, Coverdale J (2012). Survey of the prevalence of burnout, stress, depression, and the use of supports by medical students at one school. Acad Psych.

[2] Kötter T, Pohontsch NJ, Voltmer E. Stressors and starting points for health promoting interventions in medical school from the students’ perspective: a qualitative study. Perspect Med Educ. 2015;4. doi 10.1007/s40037-015-0189-5. 10.1007/s40037-015-0189-5 PMC4456465 26032519

[3] O E, McNeill K, Mavor KI, Anderson K (2014). Looking beyond personal stressors: an examination of how academic stressors contribute to depression in Australian graduate medical students. Teach Learn Med.

[4] Drolet BC, Rodgers S (2010). A comprehensive medical student wellness program—design and implementation at Vanderbilt School of Medicine. Acad Med.

[5] Dyrbye LN, Shanafelt TD (2011). Medical student distress: a call to action. Acad Med.

[6] Mavor KI, McNeill KG, Anderson K, Kerr A, O E, Platow MJ (2014). Beyond prevalence to process: the role of self and identity in medical student well-being. Med Educ.

[7] Adams J (2004). Straining to describe and tackle stress in medical students. Med Educ.

[8] Dunn LB, Iglewicz A, Moutier C (2008). A conceptual model of medical student well-being: promoting resilience and preventing burnout. Acad Psych.

[9] Bloodgood RA, Short JG, Jackson JM, Martindale JR (2009). A change to pass/fail grading in the first two years at one medical school results in improved psychological well-being. Acad Med.

[10] Reed DA, Shanafelt TD, Satele DW (2011). Relationship of pass-fail grading and curriculum structure with well-being among pre-clinical medical students: a multi-institutional study. Acad Med.

[11] Kiessling C, Schubert B, Scheffner D, Burger W (2004). First year medical students. Med Educ.

[12] Jena AB, Baldwin DC, Daugherty SR, Meltzer DO, Arora VM (2010). Presenteeism among resident physicians. JAMA.

